# Beyond urinalysis: evaluation of various clinical and laboratory reflex criteria to warrant urine culture collection in the emergency department

**DOI:** 10.1186/s12245-024-00656-8

**Published:** 2024-06-26

**Authors:** Nada M. Alateeq, Manal B. Mohammed, Albandari T. Alsubaie, Amal A. Alshehri, Dalya Attallah, Salem Agabawi, Abrar K. Thabit

**Affiliations:** 1 Department of Pharmacy Practice, Faculty of Pharmacy, King Abdulaziz University, 7027 Abdullah Al-Sulaiman Rd Jeddah, Jeddah, 22254-2265 Saudi Arabia; 2 Department of Clinical and Molecular Microbiology Laboratory, King Abdulaziz University Hospital, Jeddah, Saudi Arabia; 3 Department of Internal Medicine, Faculty of Medicine, King Abdulaziz University, Jeddah, Saudi Arabia

**Keywords:** Urinalysis, Urinary tract infection, Urine culture, Urinalysis reflex

## Abstract

**Background:**

Clinical criteria are essential for diagnosing urinary tract infections (UTIs) followed by urine testing, including urinalysis (UA). No study has evaluated the potential related factors that may guide the appropriate collection of urine cultures. Therefore, we aimed to assess the factors that may guide the appropriate collection of urine cultures.

**Methods:**

This was a case-control study of patients for whom a urine culture and a UA were ordered in the emergency department (ED) between February 2018 and December 2022. The cases included patients with positive cultures, whereas the controls included patients without growth. Patients were excluded if they were pregnant, underwent any urological procedure, received antibiotics within 3 days before ED presentation, or before culture collection.

**Results:**

Of the 263 patients, 123 had growth and 140 did not have growth in urine cultures. In the univariate analysis, female gender, urinary symptoms, urinary white blood cell (WBC) count > 5 cells/hpf, and nitrite in urine were significantly associated with growth (*P* < 0.05). However, only female gender (aOR, 1.86; 95% CI, 1.06–3.24), urinary WBC count > 5 cells/hpf (aOR, 4.60; 95% CI, 2.21–9.59), and positive nitrite in urine (aOR, 21.90; 95% CI, 2.80–171.00) remained significant in the multivariable analysis. These factors also remained significant in the subgroup of patients with urinary symptoms, except for the female gender.

**Conclusion:**

A high urinary WBC count and positive nitrite in UA should be utilized as a guide to collect urine culture, particularly in female patients, to limit the unnecessary ordering of urine culture in the ED. These factors can be used as evidence-based UA reflex criteria as an antimicrobial stewardship intervention.

## Introduction

Urinary tract infections (UTIs) are a frequent infectious disease occurring in hospitalized and non-hospitalized patients. UTIs can be either lower (cystitis) or upper (pyelonephritis) and can be either complicated or uncomplicated. Uncomplicated UTIs commonly affect women, children, and elderly patients who are immunocompetent. Complicated UTIs are consistently associated with indwelling catheters, urinary tract abnormalities, immunosuppression, or exposure to antibiotics. Various organisms can cause UTIs, the most prevalent of which are *Escherichia coli, Klebsiella pneumoniae, Proteus mirabilis, Enterococcus* spp., and *Staphylococcus saprophyticus* [1].

Clinical criteria form the basis for the diagnosis of UTI. Such clinical criteria include symptoms, such as dysuria, urinary frequency or urgency, nocturia, urinary incontinence, gross hematuria, suprapubic pain, offensive odor, and turbid urine, as well as a prior history of UTI [2].

The second essential element for the diagnosis of UTIs is urine testing, including urine analysis or urinalysis (UA), which is a rapid diagnostic tool that provides significant clinical information about urine composition, and urine microbiological cultures [2, 3]. UA can be done via either dipstick testing or microscopic examination to evaluate the presence of nitrite, white blood cells (WBCs), red blood cells (RBCs), pus, or bacteria. While UA can provide information that helps the clinician in identifying potential urinary problems, urine culture is considered the gold-standard test for diagnosing UTIs as it provides quantitative information and identification of the pathogen involved [3, 4]. In certain cases, such as complicated UTIs, urine culturing maybe repeated to ensure bacterial eradication [5].

Although no guidelines are currently available, there are some consensus recommendations for urine culture collection. These include ordering culture in the presence of signs or symptoms of UTI (such as dysuria or flank pain), automatically canceling repeat urine cultures within five days of a positive culture (during the same hospital admission and seven days for long-term care residents), requesting a urine culture when detecting an elevation in urine WBC count, notifying clinicians that high colony counts (i.e., ≥ 100,000 CFU/mL) may not represent a true infection in the absence of symptoms or signs, encouraging clinicians not to treat asymptomatic bacteriuria or mixed flora, and listing in the culture and susceptibility report only the antibiotics recommended by the guidelines to which the organism is susceptible [6]. Overall, these recommendations aim to avoid unnecessary urine cultures that often may lead to misdiagnosis of UTIs resulting in unwarranted antibiotic utilization [6].

The term UA reflex refers to the identification of certain abnormalities in UA, usually pyuria (elevated leukocytes or pus), that could reflect bacterial growth in urine cultures [7, 8]. A few studies in the literature evaluated the UA results associated with bacterial growth in urine cultures. In these studies, a urine WBC > 5 cells/hpf and a positive urinary nitrite were the most significant UA results associated with bacterial growth [3, 7, 9]. Notably, other factors, including age, gender, and blood laboratory results of WBC count and inflammatory markers were not previously assessed for their potential association with growth in urine cultures.

While some studies have assessed UA reflex criteria for urine culturing, no study was found in the literature that evaluated other potential related factors that may guide appropriate collection of urine cultures. Therefore, this study aimed to assess such factors, including UA reflex, which may become criteria for urine culturing. Additionally, no studies from Saudi Arabia have evaluated the practice of urine analysis and urine culture ordering. Hence, the current study also aimed to evaluate such practice by assessing the number of patients in whom a urine culture order may not have been necessary.

## Methods

### Study design and patients

This was a retrospective case-control study to evaluate the potential factors associated with bacterial growth in urine culture of any count in patients for whom urine culture was ordered. Patients who presented to the emergency department (ED) of King Abdulaziz University Hospital in Jeddah, Saudi Arabia between February 2018 and December 2022 and had both a UA and urine culture ordered for them were screened for study eligibility. The study protocol was approved by the Biomedical Research Ethics Unit of the Faculty of Medicine at King Abdulaziz University (reference no. 552 − 20).

Eligible patients were adults (≥ 18 years old) who had both urine culture and urine analysis collected at the time of presentation at the ED. Patients were excluded if they were pregnant, underwent any urologic procedure, received antibiotic therapy within 3 days before presenting to the ED or before culture collection (ED medication orders were evaluated for the timing of antibiotic ordering), or had growth of yeast cells in culture. Patients were divided into two groups: the cases group included patients who had positive urine cultures, whereas the matching control group included patients who did not have growth in their urine cultures.

### UA and urine culturing

In our institution, UA is processed fairly quickly at the biochemistry lab after urine specimen collection, where the results are uploaded to the patient’s electronic medical record within a few hours. Urine analysis is done on an aliquot of the undiluted urine using Iris iQ200/iChem auto-mated urine microscopy analyzer (Beckman Coulter Inc., Brea, CA, USA). On the other hand, urine cultures follow the standard microbiological processing, where the specimen undergoes serial dilution followed by plating on appropriate agar plates and incubated overnight at 37° C. When growth is observed on the plate, bacterial identification is carried out using matrix assisted laser desorption/ionization time of flight (MALDI-TOF) (bioMérieux, France), followed by antibiotic susceptibility testing using VITEK 2 system (bioMérieux, France). As such, the whole urine culture processing takes about 24 h.

### Evaluated factors for association with bacterial growth in urine cultures

The factors that were evaluated for their potential association with growth in urine culture included patient-specific factors, UA factors, and blood laboratory results factors. The patient-specific factors were age, gender, presence of urinary symptoms (as documented in the notes of the patients’ electronic medical record), and factors of complicated UTI, such as diabetes, renal impairment, presence of indwelling catheter prior to presentation to the ED, pregnancy, immunosuppression, and structural or functional abnormality of the urinary tract. UA factors included urinary WBC count, urinary pH, positive nitrite, presence of mucus, and presence of squamous epithelium. The blood laboratory results assessed included blood WBC count and inflammatory markers commonly associated with infections, namely C-reactive protein, erythrocyte sedimentation rate, and procalcitonin.

### Statistical analysis

The distribution of the continuous data was determined using the Shapiro-Wilk test of normality. As the data were not normally distributed, they were presented as median [interquartile range, IQR] and compared using the Mann-Whitney U test. Categorical variables were presented as numbers (percentages) and were compared using the Chi-square test or Fisher’s exact test as appropriate. Univariate and multivariable regression analyses were utilized to evaluate factors associated with urinary bacterial growth of ≥ 10^5^ CFU/mL in urine cultures, where factors with a *P* < 0.05 in the univariate analysis were included in the multivariable analysis model to generate adjusted odds ratios (aORs) and 95% confidence intervals (95% CI). Goodness of fit of the model was determined using Hosmer-Lemeshow test, whereas omnibus test of model coefficients was used to establish the model significance. An *a priori P* value of < 0.05 was used to determine the statistical significance. Statistical analysis was done using SPSS version 28.0 software (IBM Corp. Armonk, NY, USA). Subgroup analyses were conducted for the subgroup of patients who had urinary symptoms and for the subgroup of patients who had a urinary bacterial growth count of > 10^5^ CFU/mL. This bacterial growth count value was selected since it is the count used as a cutoff value to diagnose non-catheterized patients with UTI [5].

In order to achieve a power of 80% with an assumed 20% difference between the ratio of patients with urinary bacterial growth between the study and cases groups and an α-error probability of 5%, a sample size of 264 patients was required.

## Results

### Patients characteristics

A total of 263 patients were included in the study; 123 patients showed growth, and 140 did not show growth in urine cultures. Table 1 lists the baseline characteristics of patients, where the median [IQR] age was 55.7 [37.2–72.5] years and 59.3 [36.5–75.1] years in the no growth and growth groups, respectively (*P* = 0.433). Females represented the majority of the growth group (68.3% vs. 52.9%; *P* = 0.011), and none were pregnant. Only three patients in the whole cohort were kidney transplant recipients, one in the growth group and one in the no growth group (0.8% vs. 1.4%; *P* = 0.639). Additionally, only one patient had a ureteral stent and had no growth in urine culture. Significantly more patients in the growth group had urinary symptoms than in the non-growth group (49.6% vs. 30.7%; *P* = 0.002). The most commonly reported urinary symptoms included dysuria, urinary frequency, urinary urgency, foul smelling urine, flank pain, or suprapubic pain. Additionally, more patients in the growth group received a diagnosis of UTI according to the notes in their electronic medical records in comparison with those in the non-growth group (26.6% vs. 5.8%; *P* < 0.001). As such, the number of patients who subsequently received antibiotics in the ED was significantly higher in the growth group than in the control group (62.6% vs. 45.7%; *P* = 0.006). No significant differences were found in the remaining characteristics between the two groups.

**Table 1:** Characteristics of patients

Characteristic	No growth (*n* = 140)	Growth (*n* = 123)	*P* value
***Patient characteristics***			
Age (years)	55.7 [37.2–72.5]	59.3 [36.5–75.1]	0.433
Gender (female)	74 (52.9)	84 (68.3)	**0.011**
Kidney transplant recipient	2 (1.4)	1 (0.8)	0.639
Ureteral stent	1 (0.7)	0 (0)	0.348
Baseline temperature (°C)	37 [36.5–37.4]	37 [36–37.2]	0.272
Urinary symptoms	43 (30.7)	61 (49.6)	**0.002**
Presence of complicate UTI factor	88 (62.9)	71 (57.7)	0.396
Immunosuppression	51 (36.4)	44 (35.8)	0.912
Diabetes	10 (7.1)	7 (5.7)	0.633
Structural or functional abnormality of the UT	3 (2.1)	2 (1.6)	1.00
Renal insufficiency	3 (2.1)	2 (1.6)	1.00
Catheter prior to presentation	2 (1.4)	6 (4.9)	0.152
Diagnosed with UTI	8 (5.8)	33 (26.6)	**< 0.001**
***Laboratory values***			
Blood WBC	8.8 [6.3–13.8]	9.0 [6.6–11.8]	0.641
Blood WBC > 11,000 cells/mm^3^	58 (41.4)	40 (32.5)	0.136
C-reactive protein (*n* = 68)	69.6 [30.4– 133.8]	65 [36.4–116.3]	0.961
ESR (*n* = 39)	48 [28–84]	63 [54–94.5]	0.053
PCT (*n* = 42)	0.3 [0.1–0.9]	0.6 [0.3–3.7]	0.174

Data are presented as n (%) or median [interquartile range]

ED, emergency department; ESR, erythrocyte sedimentation rate; PCT, procalcitonin; UT, urinary tract; UTI, urinary tract infection; WBC, white blood cell

Urine culture and UA results are presented in Table 2. No significant difference was observed in the urine specimen source between the two groups whether it was a clean catch (midstream) or from a catheter (*P* = 0.692) although more than half of the specimens in both groups were obtained from midstream urine. The most commonly isolated organism among patients with urinary bacterial growth was *Escherichia coli* (*n* = 47; 38.2%), followed by *Klebsiella pneumoniae* (*n* = 20; 16.3%). A higher urine WBC count (and specifically a count of > 5 cells/hpf) and positive nitrite were significantly more common in the group of patients who had growth in their urine cultures (*P* < 0.001 for both comparisons), whereas urine pH and the presence of mucus or squamous epithelium were not different between the two groups.

**Table 2:** Urine culture and urine analysis results

Characteristics	No growth (*n* = 140)	Growth (*n* = 123)	*P* value
***Urine Culture results***			
Specimen source			0.692
Midstream	82 (58.6)	75 (61)	
Catheter	58 (41.4)	48 (39)	
Amount (CFU/mL)			N/A
100-1,000	N/A	1 (0.8)	
1,000–10,000	N/A	3 (2.4)	
10,000-100,000	N/A	24 (19.5)	
> 100,000	N/A	64 (52)	
Mixed growth	N/A	31 (25.2)	
Organism			N/A
*Escherichia coli*	N/A	47 (38.2)	
*Klebsiella pneumoniae*	N/A	20 (16.3)	
*Proteus mirabilis*	N/A	9 (7.3)	
*Enterococcus faecalis*	N/A	3 (2.4)	
*Enterococcus faecium*	N/A	3 (2.4)	
*Pseudomonas aeruginosa*	N/A	2 (1.6)	
*Acinetobacter baumannii*	N/A	2 (1.6)	
*Providencia stuartii*	N/A	1 (0.8)	
*Enterobacter aerogenes*	N/A	1 (0.8)	
*Staphylococcus aureus*	N/A	1 (0.8)	
Mixed growth	N/A	1 (0.8)	
*Staphylococcus saprophyticus*	N/A	1 (0.8)	
*Enterobacter cloacae*	N/A	1 (0.8)	
*Citrobacter freundii*	N/A	31 (25.2)	
Resistance			
No resistance	N/A	71 (60.7)	N/A
ESBL	N/A	27 (23.1)	N/A
MDR	N/A	4 (3.4)	N/A
CRE	N/A	3 (2.6)	N/A
***Urine analysis results***			
White blood cell count (cell/hpf)	11 [4–40]	120 [24–765]	**< 0.001**
White blood cell > 5 cells/hpf	91 (65)	111 (90.2)	**< 0.001**
pH	5.5 [5–6.5]	5.5 [5–6.5]	0.361
Nitrite	1 (0.7)	19 (15.4)	**< 0.001**
Mucus	23 (16.4)	18 (14.6)	0.689
Squamous epithelium	74 (52.9)	74 (60.2)	0.233

Data are presented as n (%) or median [interquartile range]

CRE, carbapenem-resistant Enterobacterales; ESBL, extended-spectrum β-lactamase; MDR, multidrug-resistant

### Regression analysis results

Results of univariate and multivariable regression analyses are shown in Table 3. In the univariate analysis, the odds of growth in urine culture were 25.39 times higher in the case of positive nitrite in UA, which was statistically significant (95% CI, 3.29-189.53; *P* < 0.001). The second factor associated with high odds of bacterial growth was urinary WBC count > 5 cells/hpf, with an OR of 4.98 (95% CI, 2.50–9.93; *P* < 0.001). Other characteristics significantly associated with growth in urine cultures were female gender (OR, 1.92; 95% CI, 1.16–3.18; *P* = 0.011) and the presence of urinary symptoms (OR, 2.22; 95% CI, 1.34–3.67; *P* = 0.002).

**Table 3:** Factors associated with bacterial growth in urine culture

Factor	OR	95% CI	*P* value	aOR	95% CI	*P* value
Gender (Female)	1.92	1.16–3.18	**0.011**	1.86	1.06–3.24	**0.030**
Urinary symptoms	2.22	1.34–3.67	**0.002**	1.73	0.99–3.01	0.051
Urine white blood cell count > 5 cells/hpf	4.98	2.50–9.93	**< 0.001**	4.60	2.21–9.59	**< 0.001**
Positive urinary nitrite	25.39	3.35-192.75	**< 0.001**	21.90	2.80–171.00	**0.003**

The multivariable regression analysis model showed a goodness of fit. In this analysis, the characteristics that remained significantly associated with bacterial growth in urine cultures were female gender (aOR, 1.86; 95% CI, 1.06–3.24; *P* = 0.030), urinary WBC count > 5 cells/hpf (aOR, 4.60; 95% CI, 2.21–9.59; *P* < 0.001), and the presence of nitrite in the urine (aOR, 21.90; 95% CI, 2.80–171.00; *P* = 0.003).

### Subgroup analysis

In the subgroup analysis of patients with urinary symptoms (*n* = 104), only urinary WBC count > 5 cells/hpf and positive nitrate in urine were significantly associated with bacterial growth in the univariate analysis. These associations remained significant in the multivariable analysis (Table 4).

**Table 4:** Factors associated with growth in urine culture in patients with urinary symptoms

Factor	OR	95% CI	*P* value	aOR	95% CI	*P* value
Urine white blood cell count > 5 cells/hpf	6.88	2.08–22.79	**< 0.001**	6.72	1.93–23.41	**0.003**
Positive urinary nitrite	10.29	1.28–82.43	**0.008**	9.92	1.16–85.08	**0.036**

Table 5 presents the results of the subgroups analysis of patients with urinary bacterial growth count of > 10^5^ CFU/mL (*n* = 64) in comparison with the patients who had no growth in their urine cultures (*n* = 140). The female gender, a urine WBC count of > 5 cells/hpf, and positive nitrite in urine were significantly associated with urinary growth of > 10^5^ CFU/mL in both the univariate and multivariable regression analyses. However, the presence of urinary symptoms was significantly associated with this outcome in the univariate analysis only. Other patient and UA factors had a *P* > 0.05 in the univariate analysis; thus, the were not included in the multivariable analysis.

**Table 5:** Factors associated with bacterial growth count of > 10^5^ CFU/mL in urine culture (*n* = 204)

Factor	OR	95% CI	*P* value	aOR	95% CI	*P* value
Gender (female)	2.47	1.29–4.71	**0.005**	2.34	1.11–4.92	**0.025**
Urinary symptoms	2.26	1.22–4.14	**0.006**	1.78	0.89–3.56	0.102
Urine white blood cell count > 5 cells/hpf	10.95	3.27–36.72	**< 0.001**	8.11	2.38–27.70	**< 0.001**
Positive urinary nitrite	32.08	4.07-252.87	**< 0.001**	23.43	2.88-190.84	**0.003**

## Discussion

While previous studies evaluated UA reflex criteria for urine culturing, this is the first study to evaluate additional potential factors besides UA that could be associated with bacterial growth in urine cultures. We found that female gender, a high urinary WBC count, and positive nitrite were the main factors associated with urinary bacterial growth. The same factors were associated with the same outcome in patients with urinary symptoms. None of the other evaluated factors, including blood WBC count and inflammatory markers were significantly associated with urinary bacterial growth. Moreover, the current study revealed that many of the ordered urine cultures may not have been necessary given the lack of indications of bacterial presence in the urine based on UA results.

Urinary WBCs are part of the body’s immune system, and their counts increase considerably in response to infection or inflammation to eradicate the causative pathogen. Some studies have demonstrated that the absence of urinary WBCs is a strong predictor of negative bacterial growth in urine cultures and vice versa [7, 9]. Hence, this suggests a strong association between high urinary WBC count and the presence of bacteria in urine. A prior study indicated that a positive nitrite test had a low sensitivity value of 0.48, a relatively high specificity value of 0.83, a positive predictive value of 0.43, and a negative predictive value (NPV) of 0.86 in predicting UTIs. The study also revealed that WBC ≥ 5/hpf had a high sensitivity in predicting UTIs and relatively high NPV (0.95) [10]. Another study of 4,130 patients demonstrated that combining the factors of a high WBC count ≥ 5 cells/hpf in urine with a positive nitrite was significantly associated with bacterial growth in urine culture (Chi-square = 516.43; *P* < 0.001). When these UA reflex criteria were implemented as a guide for urine culture ordering, the rate of urine culture orders and hence inappropriate use of antibiotics were significantly reduced by 36.1%, from 45.1 to 9% during the study period of 3 months only (*P* < 0.001) [3]. Such finding indicated that implementing UA reflex criteria for urine culturing could be an effective antimicrobial stewardship intervention In another study, the absence of leukocyte esterase and pyuria were recognized to have a strong predictive value for negative urine cultures (NPV for both ≥ 0.9) [11]. In another study by Lynch, et al., urine reflex culturing (urinary WBC count > 10 cells/hpf) was safe and simple to use in acute care, ED care, and long-term care settings, resulting in a reduction of approximately 50% in hospital cultures, where the rate of pre-intervention culture orders was 3.6 cultures per 100 days while the post-intervention rate dropped to 1.8 cultures per 100 days (*P* < 0.001) [12]. In addition to lowering microbiological expenses, this strategy probably reduced the number of patients treated for asymptomatic bacteriuria (ASB) and misidentification of catheter-associated urinary tract infections (CAUTIs) [12]. Another study by Penney et al., which included 11,322 urine specimens, found that the implementation of a stricter UA reflex criterion (only urinary WBC count ≥ 15 cells/hpf) was associated with a significant reduction in culture order rates (from 32.5 cultures per 1,000-patient days to 8.7 cultures per 1,000-patient days; *P* < 0.001), consequently resulting in improved diagnostic efficacy and reducing antibiotic prescription rates without significantly impacting patients’ outcomes [13]. Another recent study effectively addressed the issue of excessive urine culture testing in evaluating inpatient fevers, particularly for CAUTIs. Their implementation of a diagnostic stewardship program resulted in a significant 45% reduction in urine cultures conducted for inpatients, underscoring the program’s practical efficacy and impact. The intervention was a bundle encompassing staff education, criteria formulation, and implementation of audit-feedback systems. This bundled intervention addressed the problem of unnecessary urine culturing resulting in substantial monthly cost reductions ranging from $920 to $3,910. This accomplishment underscores the importance of implementing standardized criteria for urine culture requests in the healthcare sector, providing a compelling case for similar initiatives in other medical environments [14].

It has been evidenced that females are generally more likely to be affected by UTIs than males due to multiple risk factors, including shorter urethra, menopause-induced structural modification of the mucosal linings of the urethra and the vagina, frequency of sexual intercourse, menstrual cycle changes, and lower genitourinary tract morpho-dynamic alterations. However, recurrent infections without a clear cause have also been reported in non-pregnant, premenopausal women [15]. Diagnosing UTIs in elderly women can be challenging due to factors such as cognitive impairment, prevalent lower urinary tract symptoms, and high incidence of ASB. One study highlighted the necessity for a discerning biomarker in this demographic, particularly the absence of specific pyuria assessment in women aged 65 and above. The study, which included 164 participants, showed that successful differentiation of UTIs in older women from asymptomatic controls, including those with ASB, can be achieved through quantifying pyuria using automated microscopy or urine flow cytometry. Furthermore, the commonly used pyuria cutoff of 10 leukocytes/µL exhibited low specificity (36%) for UTIs in older women, prompting a need for reconsideration, but showed high sensitivity (100%). These values indicated that a urinary WBC count of > 10 cells/µL most likely reflects a positive urine culture. However, a lower pyuria value cannot rule out bacterial growth in the culture. Considering the degree of pyuria is crucial when assessing older women for UTIs as current cutoffs may result in incorrect diagnosis, which may affect patient care and antimicrobial stewardship [16].

Only patients who visited the ED were included in the current study since we noticed that many patients in our ED get a urine culture ordered for them. This observation was also reported in previous studies [3, 8, 12, 17]. The study by Lynch et al. observed a significant impact of applying UA reflex criteria for urine culturing in the ED, where this intervention resulted in a reduction of urine culture ordering by 38% from 5.4 cultures per 100 visits to 3.3 cultures per 100 visits (*P* = 0.002) [12]. Therefore, to limit such a large number of potentially unnecessary orders of urine cultures in the ED as a stewardship initiative, we aimed to investigate the factors that can help select patients for whom a urine culture order could be necessary.

Pharmacists can play a critical role in directing the medical team towards the correct method of collecting urine cultures, ultimately leading to selecting the most suitable antimicrobial therapy and reducing unnecessary antimicrobial use. A previous study demonstrated a higher rate of appropriate culture-directed antimicrobial therapy after implementing pharmacist-assisted urine culture review in the ED (*P* = 0.0001), resulting in improved clinical outcomes of patients. Furthermore, a substantial redcution in the treatment of asymptomatic bacteriuria was observed (*P* = 0.0001) [18].

Kidney transplant recipients could be at risk of microbial colonization, usually with pathogens not detected by standard microbiological culturing that typically grows aerobic bacteria and yeast. A study by Sarier et al. detected 52 organisms in 37 urinary specimens collected from 60 patients (61.7%), where the most common organisms were anaerobes (*Gardnerella vaginalis* and other obligate anaerobes), atypical bacteria (*Ureaplasma* spp. and *Mycoplasma hominis*), and cytomegalovirus [19]. In our study, only one of three kidney transplant patients had a growth in their urine cultures with *K. pneumoniae*. Furthermore, kidney transplant patients with ureteral stents are also at risk of ureteral sent colonization, mostly with *Enterococcus* spp [20]. *Enterococcus* spp. was also the most common organism isolated from positive double J stent samples collected from non-transplant recipients [21]. In that study, microbial growth was observed in 82.5% of proximal double J stent samples and 78.4% of distal double J stent samples. Such findings indicate that ureteral stents increase the risk of microbial colonization, regardless of kidney transplant status. Although our study included only one patient with a ureteral stent who had no growth in urine culture, it remains prudent for clinicians to evaluate such patients for urinary symptoms and rule out UTI.

In the subgroup of patients with urinary symptoms presumed to be diagnosed with UTI, a urine WBC count of > 5 cells/hpf and positive nitrite in the urine were consistent predictors of urinary culture growth. There were 9.72-fold increased odds of a positive culture in those with a WBC count of > 5 cells/hpf (95% CI, 1.93–23.41; *P* = 0.003) and 9.92-fold increased odds of positive culture with positive nitrite test (95% CI, 1.16–84.08; *P* = 0.036). These high odds add to the body of evidence that indicate the strong association between high WBC count and positive nitrite in the urine with bacterial growth, which can be used as UA reflex criteria in patients presenting with urinary symptoms. These two factors, as well as the female gender and the presence of urinary symptoms were significantly associated with a bacterial growth count of > 10^5^ CFU/mL, which is the cutoff value used to establish UTI diagnosis in non-catheterized patients [5].

The design of the current study has some limitations. As a retrospective study, our study was constrained by the lack of laboratory data in the medical records of some patients, which may have made it challenging to assess such measurements as factors associated with urinary bacterial growth, such as the lack of values of some inflammatory markers for some patients. This could be overcome by conducting a prospective study involving ordering laboratory tests of interest. Moreover, we acknowledge that the cutoff bacterial growth count to diagnose catheter-associated UTI is 1,000 CFU/mL in catheterized urine and > 100,000 CFU/mL in non-catheterized urine. However, we included patients who had any level of bacterial growth as we mainly aimed to evaluate the association of various factors with bacterial growth and not solely the amount needed to diagnose UTI since the diagnosis of UTI can be established in the presence of symptoms regardless of the bacterial count [22]. Lastly, about one-quarter of the patients had mixed bacterial growth. While these patients met the study’s inclusion criteria, the lack of a specific growth count limited the possibility of including such patients in the subgroup analysis involving patients with a growth count of > 10^5^ CFU/mL.

## Conclusion

According to our findings, female gender, high WBC count in urine (> 5 cells/hpf), and the presence of nitrite in urine were significantly associated with bacterial growth in urine cultures. No additional patient or UA factors were significantly associated with urinary bacterial growth including the subgroup of patients with documented urinary symptoms. Therefore, clinicians, particularly in the ED, may use high urinary WBC count and the presence of nitrite in UA, particularly in women, as an indication for urine culture collection in order to limit the ordering of the culture in the absence of these factors. Using these factors as a guide can be implemented as an antimicrobial stewardship intervention in the ED as well as in other clinical settings, such as inpatient wards, since limiting urine culture ordering could consequently reduce inappropriate antibiotic use as illustrated in previous studies.

## Data Availability

The data of the current study are available from the corresponding author upon request.

## Abbreviations

aOR: adjusted odds ratio
ASB: Asymptomatic bacteriuria
CAUTI: Catheter-associated urinary tract infection
CFU: Colony forming unit
CI: Confidence interval
ED: Emergency department
hpf: high power field
MALDI-TOF: Matrix assisted laser desorption/ionization time of flight
OR: Odds ratio
UA: Urinalysis
UTI: Urinary tract infection
WBC: White blood cell
